# Establishment of a nomogram for predicting lymph node metastasis in patients with early gastric cancer after endoscopic submucosal dissection

**DOI:** 10.3389/fonc.2022.898640

**Published:** 2022-10-28

**Authors:** Xin Zhang, Dejun Yang, Ziran Wei, Ronglin Yan, Zhengwei Zhang, Hejing Huang, Weijun Wang

**Affiliations:** ^1^ Department of Gastrointestinal Surgery, Second Affiliated Hospital of Naval Medical University, Shanghai, China; ^2^ Department of Pathology, Second Affiliated Hospital of Naval Medical University, Shanghai, China; ^3^ Department of Ultrasound, Second Affiliated Hospital of Naval Medical University, Shanghai, China

**Keywords:** lymph node metastasis, early gastric cancer, nomogram, prediction model, logistic regression

## Abstract

**Background:**

Endoscopic submucosal dissection (ESD) has been accepted as the standard treatment for the appropriate indication of early gastric cancer (EGC). Determining the risk of lymph node metastasis (LNM) is critical for the following treatment selection after ESD. This study aimed to develop a predictive model to quantify the probability of LNM in EGC to help minimize the invasive procedures.

**Methods:**

A total of 952 patients with EGC who underwent radical gastrectomy were retrospectively reviewed. LASSO regression was used to help screen the potential risk factors. Multivariate logistic regression was used to establish a predictive nomogram, which was subjected to discrimination and calibration evaluation, bootstrapping internal validation, and decision curve analysis.

**Results:**

Results of multivariate analyses revealed that gender, fecal occult blood test, CEA, CA19-9, histologic differentiation grade, lymphovascular invasion, depth of infiltration, and Ki67 labeling index were independent prognostic factors for LNM. The nomogram had good discriminatory performance, with a concordance index of 0.816 (95% CI 0.781–0.853). The validation dataset yielded a corrected concordance index of 0.805 (95% CI 0.770–0.842). High agreements between ideal curves and calibration curves were observed.

**Conclusions:**

The nomogram is clinically useful for predicting LNM after ESD in EGC, which is beneficial to identifying patients who are at low risk for LNM and would benefit from avoiding an unnecessary gastrectomy.

## Introduction

Early gastric cancer (EGC) has been defined as a lesion confined to the mucosa and/or the submucosa layer of the stomach, regardless of the lymph node metastasis (LNM) status (1). With the technical improvement of endoscopic procedures in the past decade, endoscopic resection, including endoscopic mucosal resection (EMR) and endoscopic submucosal dissection (ESD), has been accepted as the standard treatment for selected patients with negligible risk of LNM (2). Although a comparable survival rate was yielded when applied for the appropriate indication of EGC, ESD still has a risk of residual of a metastatic regional lymph node, which might translate into clinical recurrence (3, 4). Additional radical gastrectomy with lymph node dissection is recommended once a non-curative ESD has been performed (5). Therefore, determining the risk of LNM is a critical consideration when selecting a treatment option for patients with EGC (6).

Although the currently used imaging examination methods, including CT, MRI, and EUS, are able to detect the existence of enlarged regional lymph nodes, none of them can accurately determine the metastatic status of lymph nodes (7). A previous study has revealed that some pathological parameters are risk factors for LNM in EGC, including tumor size, invasion depth, histologic type, lymphatic-vascular involvement, and the presence of an ulcer (8). Afterward, different prediction models were developed to facilitate the estimation of the likelihood of LNM (9–12). However, the conclusions drawn were controversial based on the data collected from a variety of regions and ethnics (13, 14). Moreover, it is incomplete to predict the likelihood of LNM with only existing factors, and we are still far from having an optimal model.

In this study, we aim to comprehensively analyze the demographics, clinical manifestation, laboratory examination, endoscopic findings, histology, and immunochemistry of the tumor and develop a predictive model to quantify the probability of LNM in EGC, which may help guide the selection of optimal treatment modalities.

## Methods

### Patient cohort

A total of 952 patients with EGC who underwent radical gastrectomy at the Second Affiliated Hospital of Naval Medical University between January 2012 and June 2021 were retrospectively reviewed. The inclusion criteria were as follows: (1) pathologically diagnosed as EGC (pT1), (2) underwent gastrectomy with D1+/D2 lymphadenectomy, and (3) achieved R0 radical resection. The exclusion criteria were as follows: (1) GC with distant metastasis, (2) remnant GC or recurrent GC, (3) received preoperative therapy including chemotherapy and/or radiotherapy, and (4) multiple sites of primary GC.

### Data collection

The relevant clinicopathological data of each included patient were collected. The clinical characteristics of the patients included gender, age, anemia, fecal occult blood test (FOBT), preoperative levels of carcinoembryonic antigen (CEA), and carbohydrate antigen 19-9 (CA19-9). The pathological information included tumor size, tumor infiltration (lamina propria, muscularis mucosa, and submucosa), tumor location (upper, middle, and lower), Lauren classification (intestinal, diffuse, and mixed), histological grade (well, moderately, and poorly), lymphovascular invasion (LVI), and the Ki67 labeling index.

### Statistical analysis

Continuous variables were presented as means and SDs or medians and IQRs. Categorical variables are presented as frequencies and percentages. The Kolmogorov–Smirnov test was used to test the normality for continuous variables. Differences between groups were analyzed using the *t*-test for normally distributed continuous variables and the Mann–Whitney *U* test for non-normally distributed continuous variables. The chi-square and Fisher’s exact tests (when appropriate) were performed for a comparison of categorical variables. A two-sided *p*-value lower than 0.05 was considered statistically significant. LASSO regression was used to help screen the potential risk factors of LNM in EGC patients. Multivariate logistic regression was used to establish a predictive model by incorporating the screened variables, and a nomogram was then developed. The discriminative ability was measured using the area under the ROC curve (AUC). The nomogram was subjected to bootstrapping validation (1,000 bootstrap resamples) to calculate a relative corrected concordance index. Decision curve analysis (DCA) was conducted by calculating the net benefits for a range of threshold probabilities to determine the clinical usefulness of the nomogram.

Statistical analyses were performed using SPSS software for Windows (Version 22.0) and R software (Version 4.1.2). The LASSO algorithm used the “glmnet” package for calculation. The nomogram and the dynamic nomogram were developed using the packages of “rms” and “DynNom,” respectively.

## Results

### General information

A total of 952 patients of EGC were included in the data analysis, of whom 170 patients (17.9%) had LNM and 782 patients (82.1%) had no LNM. Overall, the two groups were not balanced with regard to the characteristics except age and tumor location. The percentage of female patients was higher in the LNM group than that in the non-LNM group. The patients with LNM showed higher incidence of abnormally elevated CEA and CA19-9 levels, positive FOBT, presence of anemia, and presence of LVI. A larger tumor size, a deeper tumor infiltration, and a higher Ki67 labeling index were also observed in the LNM group. A detailed comparison of demographics, serum CEA and CA19-9 levels, and tumor pathological characteristics between the LNM and non-LNM groups is shown in **Table 1**.

**Table 1:** Characteristics of early gastric cancer patients in LNM Set and Non-LNM Set.

	LNM (n=170)	Non-LNM (n=782)	P value
Gender (Male)	99 (58.2%)	555 (71.0%)	0.001
Age (year)	60 (51-67)	60 (52-66)	0.635
CEA (<5)	134 (78.8%)	738 (94.4%)	<0.001
CA199 (<39)	155 (91.2%)	773 (98.8%)	<0.001
FOBT (positive)	59 (34.7%)	88 (11.3%)	<0.001
Anemia (positive)	64 (37.6%)	179 (22.9%)	<0.001
Lauren classification			<0.001
Intestinal	60 (35.3%)	375 (48.0%)	
Diffuse	67 (39.4%)	300 (38.4%)	
Mixed	43 (25.3%)	107 (13.7%)	
Tumor location			0.270
Lower	145 (85.3%)	625 (79.9%)	
Upper Middle	12 (7.1%) 13 (7.6%)	73 (9.3%) 84 (10.7%)	
Histological grade			<0.001
Well	7 (4.1%)	138 (17.6%)	
Moderately	52 (30.6%)	283 (36.2%)	
Poorly	111 (65.3%)	361 (46.2%)	
LVI (positive)	24 (14.1%)	12 (1.5%)	<0.001
Tumor size (cm)	2.2 (1.6-3.0)	2.0 (1.5-3.0)	0.001
Tumor infiltration			<0.001
Lamina propria	20 (11.8%)	273 (34.9%)	
Muscularis mucosa	23 (13.5%)	192 (24.6%)	
Submucosa	127 (74.7%)	317 (40.5%)	
Ki67 labeling index (%)	60 (40-70)	45 (30-60)	<0.001

LNM, lymph node metastasis; FOBT, fecal occult blood test; LVI, lymphovascular invasion.

### Nomogram development

**Figure 1:**
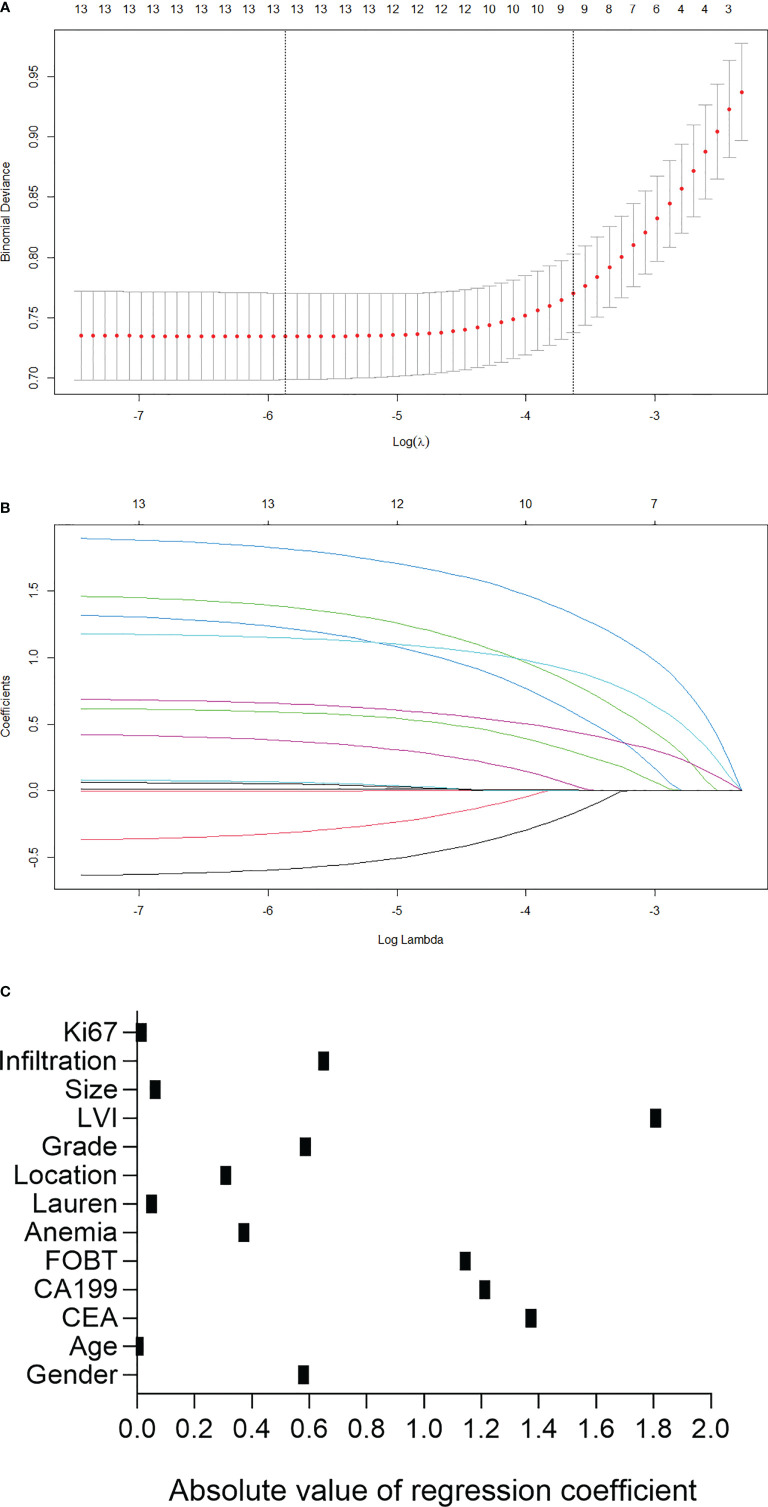
Predictor selection using the least absolute shrinkage and selection operator (LASSO) logistic regression model. **(A)** Identification of the optimal penalization coefficient lambda in the Lasso model using 10-fold cross-validation and the minimum criterion. **(B)** Lasso coefficient profiles of the 13 clinicopathological features. **(C)** Scatter diagram to show the absolute value of regression coefficient for each variable as calculated in LASSO logistic regression model. LVI, lymphovascular invasion; FOBT, fecal occult blood test.

**Figure 2:**
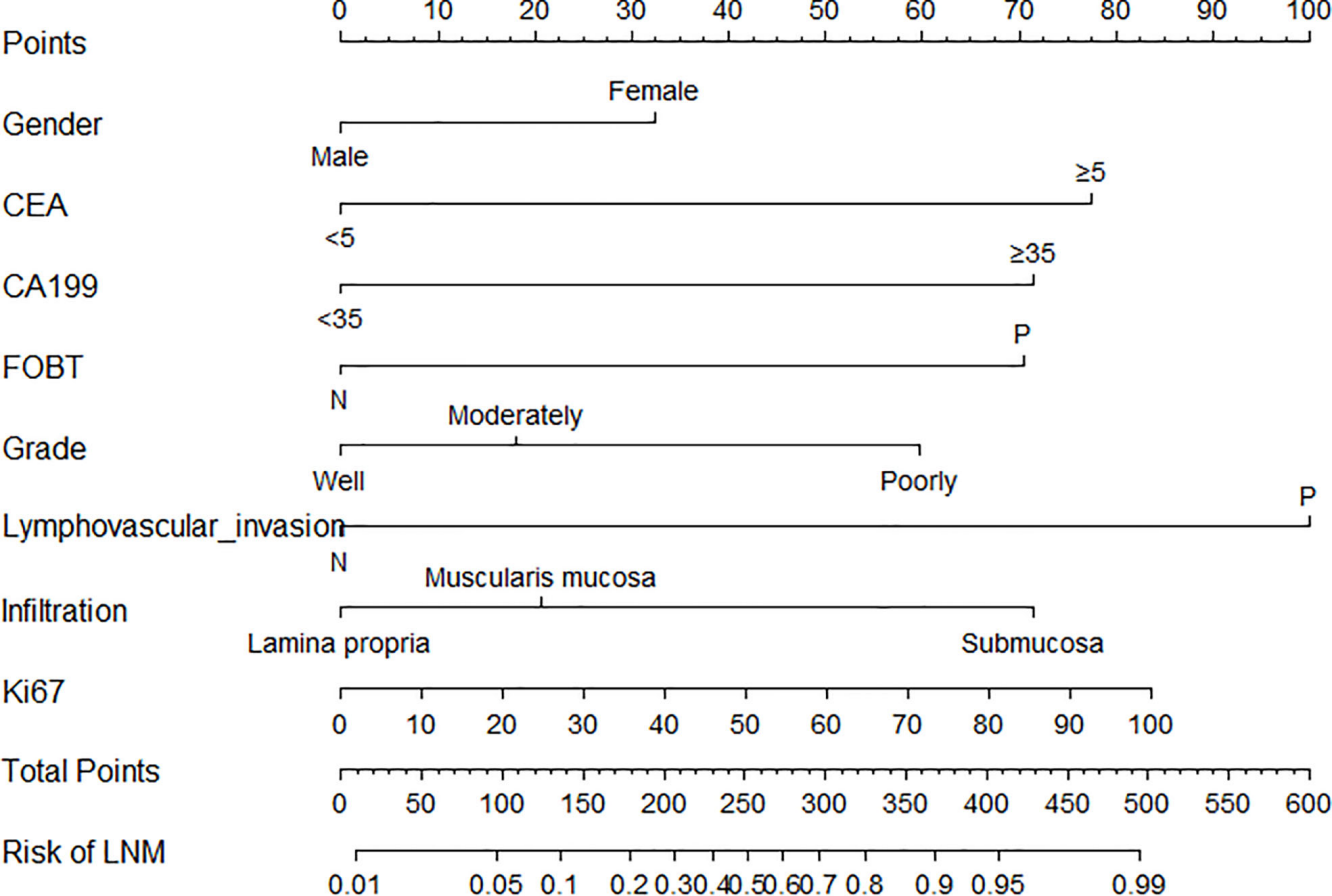
Nomogram for the prediction of lymph node metastasis after endoscopic submucosal dissection in early gastric cancer. LVI, lymphovascular invasion; FOBT, fecal occult blood test.

The parameters were firstly screened by using a LASSO-penalized binary logistic regression model. As shown in **Figure 1A**, all the 13 clinicopathological characteristics were selected to be the potential parameters. For each parameter, a coefficient profile plot was produced against the log (lambda) sequence (**Figure 1B**). It should be noted that the absolute values of the coefficients for age, tumor size, Lauren classification, and Ki67 were very close to zero (**Figure 1C**). The results of the multivariate logistic analysis revealed a total of eight independent risk-predictive factors for LNM (**Table 2**). A nomogram was developed to determine the influence of the eight variables on LNM (**Figure 2**). LVI demonstrated the most extended scale and the most significant effect on LNM.

**Table 2:** Multivariate analysis of the factors affecting LNM.

Variables (ref)	β	OR (95% CI)	P value
Gender (Female)	-0.676	0.508 (0.334-0.774)	0.002
Age	-0.007	0.993 (0.974-1.012)	0.459
CEA (<5)	1.511	4.529 (2.486-8.251)	<0.001
CA199 (<39)	1.399	4. 049 (1.360-12.056)	0.012
FOBT (Negative)	1.216	3.375 (2.115-5.385)	<0.001
Anemia (Negative)	0.411	1.509 (0.982-2.317)	0.060
Lauren (Intestinal)	ref		0.447
Diffuse	-0.458	0.632 (0.277-1.444)	0.277
Mixed	-0.223	0.800 (0.331-1.933)	0.621
Tumor location (Lower)	ref		0.076
Upper	-0.553	0.575 (0.267-1.239)	0.158
Middle	-0.687	0.503 (0.248-1.020)	0.057
Histological grade (Well)	ref		0.017
Moderately	0.336	1.400 (0.572-3.423)	0.461
Poorly	1.437	4.209 (1.360-13.028)	0.013
LVI (Negative)	1.901	6.694 (2.879-15.562)	<0.001
Size	0.099	1.104 (0.957-1.274)	0.173
Infiltration (Lamina propria)	ref		<0.001
Muscularis mucosa	0.378	1.459 (0.751-2.836)	0.265
Submucosa	1.284	3.613 (2.053-6.355)	<0.001
Ki67 labeling index (%)	0.016	1.016 (1.006-1.025)	0.001

LVI, lymphovascular invasion; FOBT, fecal occult blood test.

The nomogram applications are described as follows: the sum of the total scores can be obtained by adding each score of the corresponding variable. The predictive risk corresponding to the total score is the risk of LNM in EGC. Moreover, a practical and accurate online dynamic nomogram was established (**Supplementary Figure S1**), which could be obtained by visiting https://hanl10.shinyapps.io/DynNomapp/.

### Discrimination and calibration

**Figure 3:**
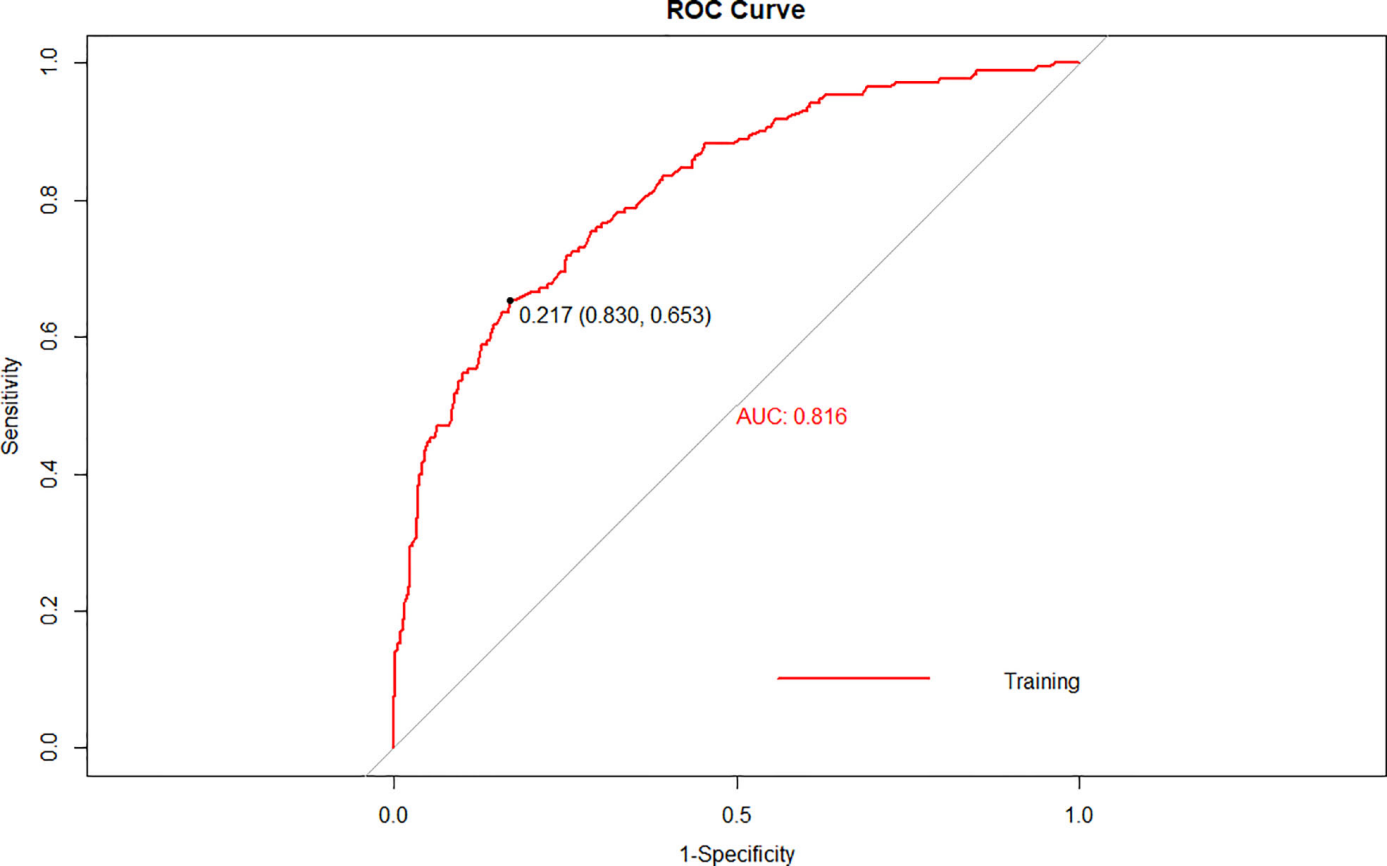
A receiver operating characteristics (ROC) curve of the multivariate logistic regression model for the prediction of lymph node metastasis after endoscopic submucosal dissection in early gastric cancer.

**Figure 4:**
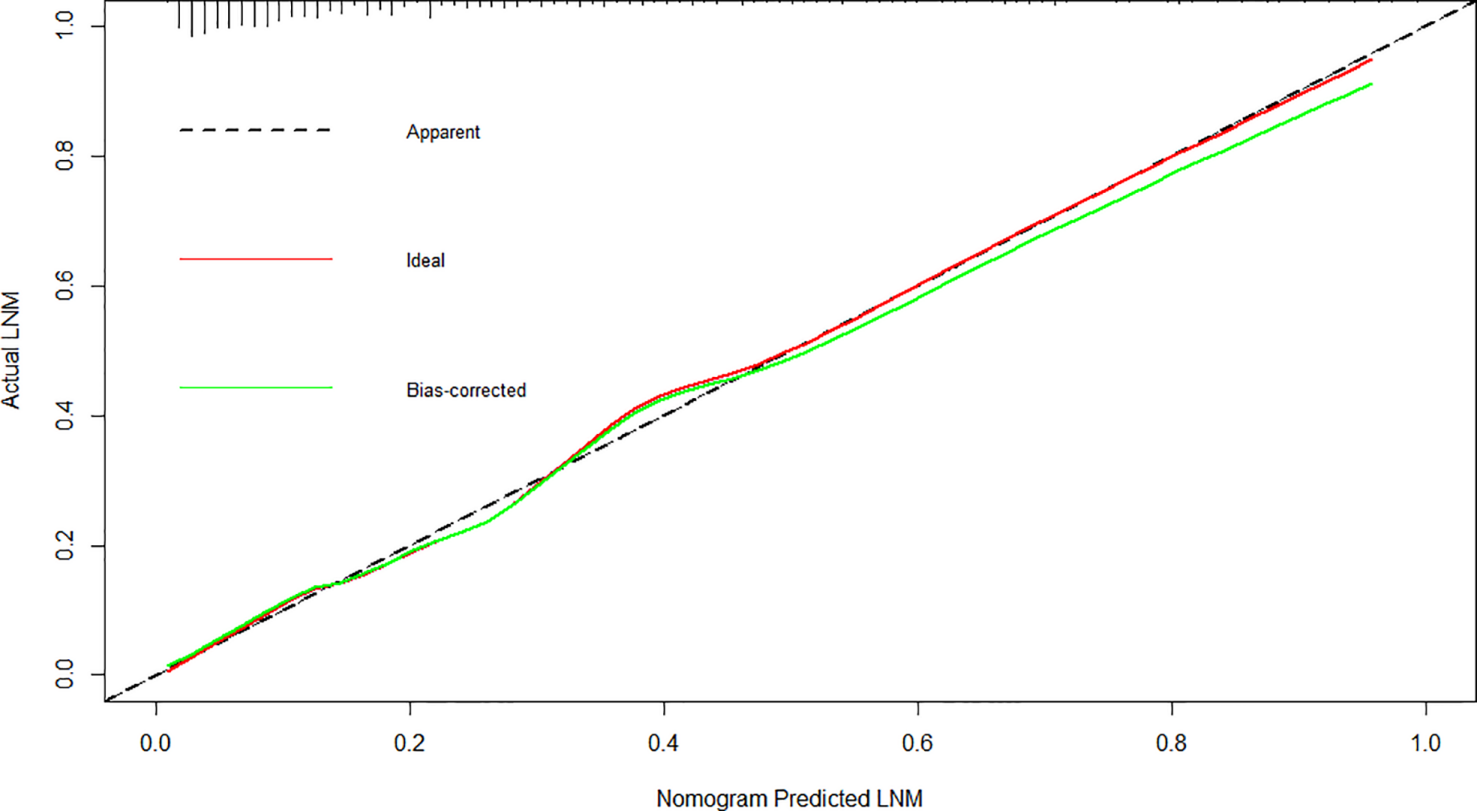
Calibration plot for the nomogram for the prediction of lymph node metastasis after endoscopic submucosal dissection in early gastric cancer.

The cutoff value of the predictive nomogram was 183.8 when the maximum of the concordance index (AUC) reached 0.816 (95% CI 0.781–0.853) (**Figure 3**). The sensitivity, specificity, positive predictive, and negative predictive values of the nomogram were 0.653, 0.830, 0.455, and 0.917, respectively. To verify the accuracy of the model, a corrected concordance index was calculated using the 1,000 bootstrap resample method, and the value of 0.805 (95% CI 0.770–0.842) suggested that the established nomogram had a high accuracy in discriminating the LNM. Moreover, high agreements between ideal curves and calibration curves were observed (**Figure 4**).

### Clinical application

**Figure 5:**
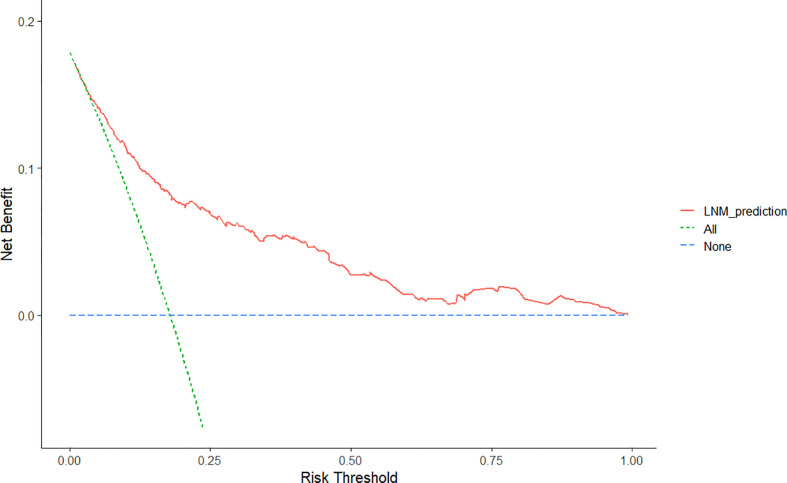
Decision curve analysis for the nomogram for the prediction of lymph node metastasis after endoscopic submucosal dissection in early gastric cancer. The y-axis represents net benefits, calculated by subtracting the relative harms (false positives) from the benefits (true positives). The x-axis calculates the threshold probability.

Furthermore, the DCA for the nomogram showed that there is a net benefit by using this nomogram for predicting LNM in EGC patients when the predicted probability of LNM was between 6% and 99%, suggesting that the nomogram was clinically useful (**Figure 5**).

## Discussion

With the popularization of endoscopic screening, an increasing number of EGCs were detected (15, 16). On the whole, the prognosis of EGC is favorable, with a 5-year overall survival rate exceeding 95% (4). Although gastrectomy with concomitant lymphadenectomy was considered the gold standard treatment for EGC, radical surgery is associated with high morbidity and mortality rates, as well as a decrease in the quality of life (17, 18). Therefore, minimizing the amount of invasive procedures applied in the treatment of EGC has become a matter of concern in dealing with GC patients. In the past decades, the ESD procedure was technically established, and the clinical utility of long-term outcomes for possible node-negative EGC has also been confirmed in both Eastern and Western populations (19, 20). However, considering that the pretreatment diagnosis could be incorrect for 20% of the tumors that are candidates for endoscopic resection (21), it is essential to make an accurate curability assessment after ESD to avoid non-curability of such early lesions.

For each patient who has undergone ESD, the final pathology is routinely reviewed to determine whether a negative margin has been achieved and, more importantly, the risk of LNM. Currently, Japanese guidelines indicate the curability after ESD for EGC based on the eCura system (22). In the past few years, the clinical value of the eCura system was confirmed for predicting the risk of LNM and guiding treatment selection after ESD (23, 24). However, the eCura system does not provide a quantified estimation, and the treatment recommendation for intermediate-risk patients is not as clearly determined. Moreover, the eCura system includes only pathological data, which may lead to insufficient predictive power of the model. Therefore, we have developed a novel nomogram used for the prediction of LNM in EGC patients who have undergone ESD, which may help identify patients who are at low risk for LNM and would benefit from avoiding an unnecessary gastrectomy. In addition, this model can provide a quantitative prediction value of the risk of LNM for each individual, which might serve as an objective reference for the patients themselves to participate in the decision-making of the following treatment.

Although there are many published relevant studies on the prediction of LNM in EGC, the exploration for the optimal model has never ended (12, 25–30). Of particular note, Chen et al. put forward a model by incorporating the collagen features of the tumor tissues extracted from multiphoton imaging, which showed a more robust ability to estimate the risk of LNM than traditional models (10). However, the procedure has not been routinely used in clinical practice due to their complicated operation and high cost. In this study, we firstly performed a variable screening using the LASSO Cox regression method from the data indicators generated during the patients’ routine visit. Then, multivariate logistic analysis identified a total of eight independent risk-predictive factors for LNM, and a nomogram was developed accordingly. In comparison with the previous studies (11, 12, 27, 29, 30), this model showed relatively high accuracy in discriminating LNM in EGC, with an AUC of 0.816 (95% CI = 0.781–0.853) in the training set and 0.805 (95% CI = 0.770–0.842) in the validation set by using the bootstrap resample method. Second, this nomogram was well calibrated in the development and validation cohorts, which further confirmed the predictive stability of its clinical implications. Moreover, the dynamic nomogram is accessible online, which facilitates the rapid and accurate calculation of the risk estimation. The nomogram has a high negative predictive value (0.917), which means that it has a high accuracy to identify patients who might not have LNM and hence not need gastrectomy. However, the low-presented positive predictive value (0.455) indicated that a decision of gastrectomy based on this predictive model needs to be made with caution.

Three traditional histopathological parameters were included in our nomogram model. LVI usually indicates the presence of LNM or, at least, lymph node micrometastasis (31), and some researchers even equate LVI with LNM (32). Lee et al. (33) developed a modified eCura system by dividing the factors into LVI and other factors, where LVI was even given a higher weight, to predict the risk of LNM. In our present study, the LNM rate was 15.9% among patients without LVI but as high as 66.7% in patients with LNM. Therefore, LVI is deemed as the predominant predictor for LNM, which is in accordance with most previous studies (9, 34). Numerous studies have indicated that the depth of tumor invasion and the tumor differentiation grade have great impact on LNM (11, 35, 36). Paralleled with these studies, we also confirmed that the two variables are important independent risk factors for LNM. Nevertheless, because the results of these three pathological parameters cannot be precisely evaluated without the entire resected specimen, this nomogram is applicable only to patients with EGC who underwent complete endoscopic resection and not to those aiming to perform preoperative evaluation.

The incidence of GC is known to be higher in men than women, but female patients seem to have a worse prognosis (37, 38). In line with previous studies, our data also reveal that female patients have a higher risk of LNM than male patients (23.8 *vs.* 15.1%). A study by Huang et al. (39) revealed that the elevated plasma levels of tumor biomarkers CEA and CA19-9 were associated with the increased incidence of LNM in EGC. Consistently, the present study also confirmed this conclusion, and the two tumor biomarkers were included in the nomogram model.

The FOBT has been widely used to screen for gastrointestinal tumors, including gastric cancer and colorectal cancer (40). Previous studies have shown that the all-cause mortality and the non-colorectal cancer mortality of patients with FOBT positivity are significantly increased (41). Lu et al. (42) showed that the overall postoperative complications were significantly higher in the preoperative FOBT-positive group than those in the preoperative FOBT-negative group, and the FOBT was an independent risk factor for 5-year overall survival, implying that FOBT results may have a more prognostic value. In the present study, patients with a positive result of the FOBT showed a higher risk of LNM than those with a negative result (40.1% *vs.* 13.8%). It is the first time to report that FOBT results have a predictive value for the LNM of EGC. Although the results of the FOBT may be affected by some other digestive tract disorders, especially perianal diseases, which may cause false positives, these results can be used as background data for potential future large-scale, multicenter clinical studies to further validate the predictive value of the FOBT for LNM.

Regarding the relationship between the ki67 labeling index and LNM in GC, previous studies have shown inconsistent findings. The Ki67 labeling index was recognized as an important predictor for predicting the LNM in gastric cancer in two earlier studies (43, 44). In contrast, there were also studies showing that there was no relationship between immunohistochemical staining for Ki67 and LNM (45–47). In the present study, our data showed that the Ki67 labeling index was an independent risk factor for LNM in EGC, and it is the only continuous variable incorporated in the nomogram model. These results indicate that the measurement of Ki67 could be a useful additional parameter in the assessment of indications for supplemental gastrectomy after ESD.

Some limitations exist in this study. First, external validation using data from other institutions or populations was not performed. Concern about generalization may be warranted. Second, the patients included in this study are all Chinese, and most of them resided in the coastal area of East China. Therefore, the generalizability of the findings to populations with different races, ethnics, or geographical environments may be limited. Finally, the diagnosis of LNM was based on conventional hematoxylin and eosin staining in this study, which may result in the omission of lymph node micrometastasis. Because it is still unclear about the incidence or clinical significance of lymph node micrometastasis, selecting patients for radical gastrectomy by using this model might, to some extent, affect prognoses.

In conclusion, we detail a risk-prediction model based on the comprehensive clinicopathological examination for predicting LNM in EGC. As there are no definitive criteria to identify patients with EGC who have a low risk for LNM, this model may be useful in clinical practice because it may facilitate the more accurate selection of patients who can undergo ESD without additional surgery.

## Data availability statement

The raw data supporting the conclusions of this article will be made available by the authors, without undue reservation.

## Ethics statement

The studies involving human participants were reviewed and approved by the ethics committee of Shanghai Changzheng Hospital. The patients/participants provided their written informed consent to participate in this study. Written informed consent was obtained from the individual(s) for the publication of any potentially identifiable images or data included in this article.

## Author contributions

XZ and WW: designed this study. DY and ZW: acquired and analyzed the data. XZ and HH: wrote and edited the manuscript. All authors contributed to the article and approved the submitted version.

## Funding

This study was supported by grants from the National Natural Science Foundation of China (81773049, 81402359,and 81602617).

## Acknowledgments

The authors would like to thank Ms Li Han, Manager of the Department of the Clinical Data Management, Roche Asia Pacific R&D Center, for her help and guidance in the programming and deploying of the dynamic nomogram.

## Conflict of interest

The authors declare that the research was conducted in the absence of any commercial or financial relationships that could be construed as a potential conflict of interest.

## Publisher’s note

All claims expressed in this article are solely those of the authors and do not necessarily represent those of their affiliated organizations, or those of the publisher, the editors and the reviewers. Any product that may be evaluated in this article, or claim that may be made by its manufacturer, is not guaranteed or endorsed by the publisher.
