# Supplementary material for: Establishment of a nomogram for predicting lymph node metastasis in patients with early gastric cancer after endoscopic submucosal dissection
Source: Front Oncol. 2022 Oct 28;12:898640. doi: 10.3389/fonc.2022.898640 (PMC9651963; doi:10.3389/fonc.2022.898640)

Supplementary Figures

Figure S1. Screenshot of the practical utility of the online dynamic nomogram for the prediction of lymph node metastasis after endoscopic submucosal dissection
FOBT, fecal occult blood test

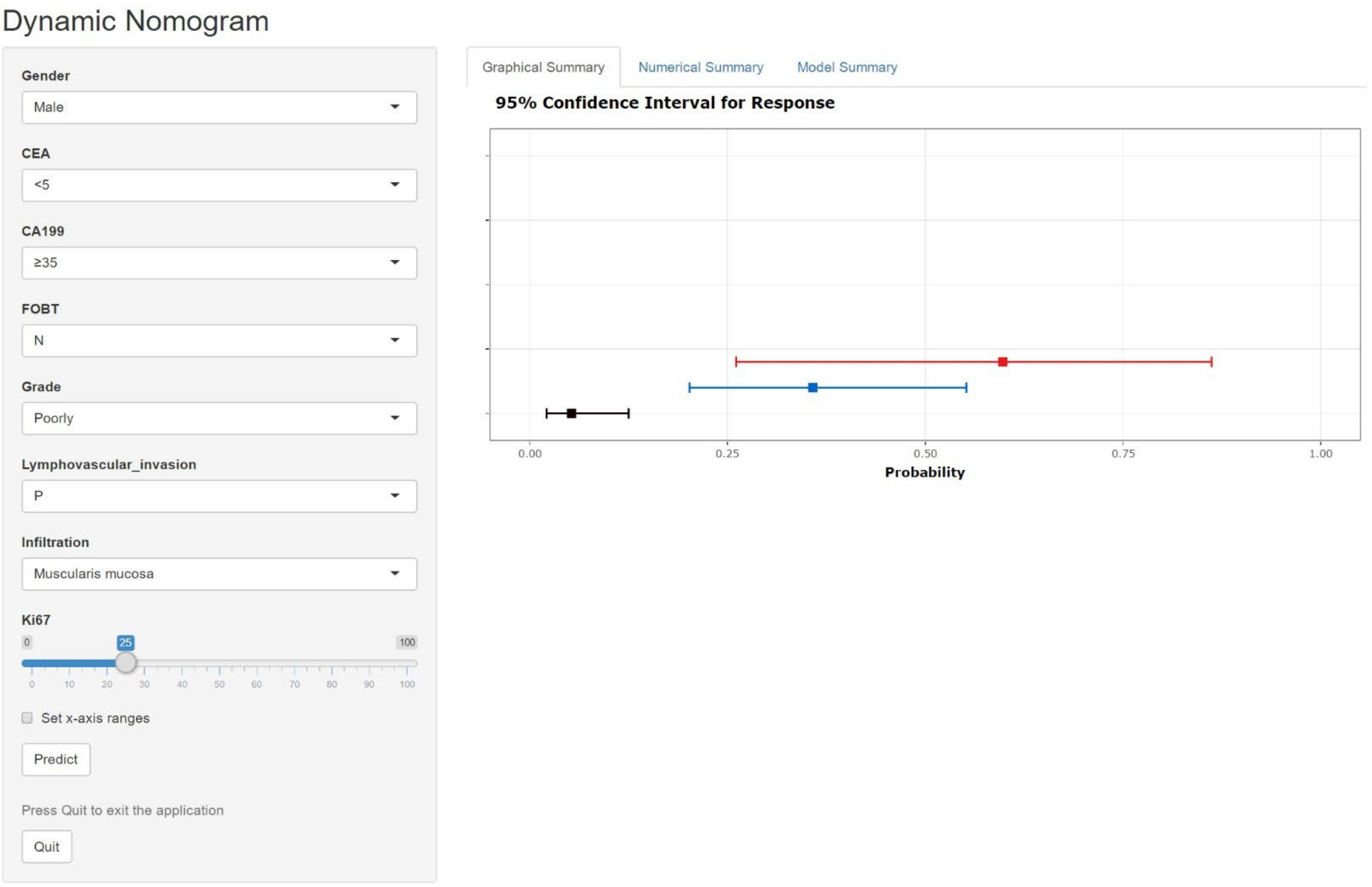

Supplement: Supplementary file 1 [file DataSheet_1.pdf]
